# Recidivism at the puerto rico trauma hospital

**DOI:** 10.1007/s00068-020-01487-x

**Published:** 2020-09-18

**Authors:** Adriana Suárez-Cruz, Ediel O. Ramos-Meléndez, Mariely Nieves-Plaza, Lourdes Guerrios, Pablo Rodríguez-Ortiz

**Affiliations:** Trauma Surgery Div, Dept. of Surgery, School of Medicine, University of Puerto Rico Medical Sciences Campus, San Juan, PR USA

**Keywords:** Recidivism, Reinjury, Recurrence, Trauma

## Abstract

**Purpose:**

Although trauma represents a leading cause of morbidity and mortality worldwide, there is limited and heterogeneous evidence regarding trauma recidivism and its outcomes. This analysis determined the rate and independent risk factors of trauma recidivism and compared the first and second injury episode among recidivists.

**Methods:**

An IRB-approved retrospective cohort study was performed with data from the Puerto Rico Trauma Hospital Registry. Bivariate analyses were done using Pearson’s Chi squared, Wilcoxon rank-sum, McNemar, Stuart-Maxwell or Wilcoxon signed-rank tests, as appropriate. Independent predictors for recidivism were determined through a logistic regression model. Statistical significance was set at *p* < 0.05.

**Results:**

24,650 patients were admitted to the hospital during 2000–2017. Recidivism rate was 14 per 1,000 patients discharged alive. Males and individuals aged 15–24 years old were 3.88 (95% CI: 2.21–6.80) and 3.80 (95% CI: 2.24–6.46) times more likely to be recidivists, respectively. Contrariwise, an ISS $\ge$ 25 [adjusted odds ratio (AOR) = 0.44; 95% CI: 0.28–0.68] and a GCS $\le$ 8 (AOR = 0.56; 95% CI 0.34–0.92) were protective factors. Furthermore, recidivists exhibited less in-hospital mortality than their non-recidivist counterparts (7.2% vs. 10.7%; *p* = 0.045).

For recidivists, the median (interquartile range) time to reinjury was 42 (59) months; and the second injury episode was more severe than the first one, as the proportion of patients with ISS $\ge$ 25 increased (7.9% vs. 14.1%; *p* = 0.022).

**Conclusion:**

The independent predictors of trauma recidivism and the median time to reinjury identified in this study provide valuable information to the development of prevention strategies aimed at reducing the burden of injury.

## Introduction

Trauma kills over five million people globally in a year, generating 9% of the world’s mortality [1]. In the United States (US), trauma contributes to two of the ten leading causes of death—unintentional injuries and suicides—and the leading cause of death in individuals aged 1–44 years old [2]. Non-fatal injuries represent 68% ($456.9 billion dollars) of all medical and life productivity loss costs; while fatal injuries account for nearly 33% of all medical and life productivity loss [3, 4].

There is limited data regarding a rare population of trauma patients that present an injury on two or more separate occasions (recidivists). The rates of recidivism vary largely, ranging from 0.38% to 44% depending on location [5, 6]. Several characteristics including sex, race, socioeconomic position (neighborhood level), substance use, health insurance status, employment status, and civil status have been associated with recidivism [5–9]. In addition, a couple of studies reported recidivists were likely to present the same mechanism of traumatic injury in a subsequent admission as in the first injury [5, 10]. However, due to the heterogeneous classification of mechanisms of injury, the prevalence and trends of mechanisms associated with recidivism remain unclear.

Moreover, when comparing recidivists to non-recidivists, there have been either contradictory findings or little findings regarding the outcome variables—the Injury Severity Score (ISS), the Glasgow Coma Scale (GCS), the hospital length of stay (LOS), the amount of days in the trauma intensive care unit (TICU), the requirement for mechanical ventilation (MV), the MV days, and mortality [5, 8, 12, 13]. The window for reinjury has been estimated to vary largely between 7.9 months and 45 months [5, 14]. Defining time to reinjury among recidivists is instrumental due to its implications in prevention efforts.

Puerto Rico (PR) is an unincorporated territory of the US, with a largely predominant Hispanic population. In 2016, about 43.5% of the population was below the poverty level, more than triple the national average of the US and double the poorest state in the US‒Mississippi [15]. The Puerto Rico Trauma Hospital (PRTH) attends all levels of injury-related medical emergencies, being the only tertiary level II trauma center in PR. The hospital offers a unique setting to evaluate factors that may predispose to trauma recidivism. Thus, the present study aimed to determine the rate and independent risk factors of trauma recidivism, as well as comparing clinical and injury-specific features of the first and second traumatic event in recidivist patients. Findings could encourage policy advancement, and the development of tailored cost-effective intervention programs at hospital and community level.

## Methods

### Study design and setting

We conducted a historical cohort study of patients admitted to the PRTH. This level II trauma center is the major referral hospital for polytrauma patients in PR and the Caribbean. Furthermore, our center participates in the US National Trauma Registry System, which allows patient data to be collected in a standardized way and, in turn, facilitates comparisons with other trauma centers nationwide. The trauma registry consists of information transcribed directly from medical records, and is subject to a quarterly quality-control review, conducted under the standards developed by the American College of Surgeons, providing credible and official data for the present study.

### Study population

Between April 2000 and June 2017; 24,650 patients with first-time injuries were treated in our institution and included in this research. Once each patient was discharged, their follow-up period was started to determine trauma recidivism. The observation period was extended until December 2017, to ensure a minimum 6-month follow-up for all patients by the end of study. Trauma recidivism was defined as subsequent admissions to our hospital owing to a new injury episode. Patient’s name, date of birth, and medical record number were used to identify trauma recidivists. Admissions associated to follow-up treatment or complications for a previous injury were excluded.

### Variables

Sociodemographic characteristics of interest were sex, age, health insurance coverage, and health region where the patient lives (based on the eight Puerto Rico Government Health Plan regions). Moreover, we considered the following injury-related and clinical data: mechanism of injury, type of injury, arterial base deficit (ABD), breathing, systolic blood pressure (SBP), temperature, heart rate, ISS, and GCS. All clinical parameters were recorded upon admission to the hospital. Data on hospital course and outcomes included TICU admission, hospital and TICU LOS, need for MV, days on MV, and in-hospital mortality. For recidivist trauma patients, time to reinjury was also considered.

### Statistical analysis

Trauma recidivism rate at the PRTH for the 2000–2017 period was determined by dividing the number of recidivist patients by the total of patients discharged from the hospital alive (i.e., those with first-time injuries who died in the hospital were excluded from the denominator in computing this rate).

When comparing sociodemographic characteristics, injury profile, hospital course, and outcomes of trauma patients according to their recidivism status, all subjects were included to take into account the full spectrum of disease severity (i.e., those with first-time injuries who died in the hospital were considered). Contrasts of categorical and continuous data between recidivists and non-recidivists were performed through Pearson’s Chi squared test and Wilcoxon rank-sum test, respectively. These between-group differences were assessed using data from the patients' first admission, except for in-hospital mortality, where the last admission was used. Furthermore, an unconditional logistic regression analysis was done to determine the factors independently associated with trauma recidivism. Our *p* value criterion for entering variables into the model was set at 0.05. However, due to the large number of variables considered, a more restrictive significance level (*p* < 0.01) was used to assess first-order interaction terms (i.e., all possible pairwise interactions). This would allow us to include only strong interaction effects, which would be misleading to suppress [16].

Finally, the sub-analysis comparing injury-related and hospital-based factors between the first and second injury episode in recidivist trauma patients were conducted using McNemar test or Stuart-Maxwell test for categorical variables, and Wilcoxon signed-rank test for continuous ones.

The threshold probability for statistical significance was set at 0.05, except for interaction effects. All data were analyzed using STATA version 14 (STATA Corp, College Station, TX, USA). This study was approved by the Institutional Review Board of the Medical Sciences Campus of the University of Puerto Rico.

## Results

A total of 24,650 patients were admitted to the PRTH during the study period. The rate of trauma recidivism was 14 per 1000 patients discharged alive. Table 1 depicts the comparison of sociodemographic characteristics, injury profile, hospital course, and outcomes between the study groups. Recidivists were predominantly male (95.4% vs. 82.6%; *p* < 0.001), and frequently within the 15- to 24-year-old age range (40.1% vs. 25.4%; *p* < 0.001) when compared to non-recidivists. Furthermore, those patients with more than one trauma-related admission were mostly from Metro-North (25.2% vs. 20.3%), San Juan (18.6% vs. 15.0%), East (17.3% vs. 16.3%), and Northeast (14.1% vs. 13.7%) Health Regions when compared to their counterparts with only one trauma-related admission (*p* = 0.013). Additionally, the proportions of patients with public health insurance (39.4% vs. 26.5%) or uninsured (12.3% vs. 10.6%) were higher in the recidivism group than in the non-recidivism group (*p* < 0.001).

**Table 1:** Comparison of Sociodemographic Characteristics, Injury Profile, Hospital Course, and Outcomes between Recidivist and Non-Recidivist Trauma Patients Admitted to the Puerto Rico Trauma Hospital

Characteristic	Recidivist (*n* = 307) *n* (%)	Non-Recidivist (*n* = 24,343) *n* (%)	*p* value	
Sociodemographic data				
Sex			< 0.001	
Male	293 (95.4)	20,091 (82.6)		
MD	(*n* = 0)	(*n* = 7)		
Age, years			< 0.001	
Median (IQR)	27 (20)	33 (28)		
Categories			< 0.001	
< 15	9 (2.9)	1437 (5.9)		
15—24	123 (40.1)	6185 (25.4)		
25—34	70 (22.8)	5385 (22.1)		
35—44	46 (15.0)	3566 (14.7)		
45—54	40 (13.0)	2921 (12.0)		
55—64	14 (4.6)	2199 (9.0)		
> 64	5 (1.6)	2650 (10.9)		
Health region			0.013	
West	17 (5.6)	2351 (9.8)		
North	25 (8.2)	2495 (10.4)		
Metro-north	77 (25.2)	4870 (20.3)		
San Juan	57 (18.6)	3602 (15.0)		
Northeast	43 (14.1)	3297 (13.7)		
Southwest	12 (3.9)	1419 (5.9)		
Southeast	22 (7.2)	1763 (7.3)		
East	53 (17.3)	3926 (16.3)		
Other (no PR)	0 (0)	303 (1.3)		
MD	(*n* = 1)	(*n* = 317)		
Insurance status			< 0.001	
Private insurance	141 (48.3)	14,744 (62.9)		
Public insurance	115 (39.4)	6204 (26.5)		
Uninsured	36 (12.3)	2482 (10.6)		
MD	(*n* = 15)	(*n* = 913)		
Injury-related data				
Mechanism of injury			< 0.001	
MVA	108 (35.2)	8961 (36.8)		
GSW	93 (30.3)	4631 (19.0)		
SW	29 (9.5)	1884 (7.7)		
Falls	33 (10.8)	3870 (15.9)		
Pedestrians	14 (4.6)	2815 (11.6)		
Others	30 (9.18)	2179 (9.0)		
MD	(*n* = 0)	(*n* = 3)		
Type of injury A			< 0.001	
Penetrating	121 (39.5)	6,544 (26.9)		
MD	(*n* = 1)	(*n* = 21)		
Type of injury B			< 0.001	
Violence related	137 (44.6)	7149 (29.4)		
Fall	33 (10.8)	3870 (15.9)		
Motor vehicle related	122 (39.7)	11,776 (48.4)		
Others	15 (4.9)	1545 (6.3)		
MD	(*n* = 0)	(*n* = 3)		
ABD, mEq/L			0.304	
< −2	72 (48)	7853 (54.3)		
−2—2	63 (42)	5306 (36.7)		
> 2	15 (10)	1306 (9.0)		
MD	(*n* = 157)	(*n* = 9878)		
Breathing, bpm			0.085	
< 12	0 (0)	298 (1.3)		
12—20	193 (65.9)	14,470 (62.0)		
> 20	100 (34.1)	8553 (36.7)		
MD	(*n* = 14)	(*n* = 1022)		
SBP, mmHg			0.136	
Median (IQR)	130 (27)	126 (32)		
MD	(*n* = 2)	(*n* = 259)		
Temperature, F			0.155	
< 95.1	10 (3.3)	1326 (5.7)		
95.1 F—100.8	291 (96.0)	21,854 (93.2)		
> 100.8	2 (0.7)	257 (1.1)		
MD	(*n* = 4)	(*n* = 906)		
Heart rate, bpm			0.177	
< 60	18 (5.9)	1150 (4.8)		
60—100	194 (63.4)	14,449 (59.7)		
> 100	94 (30.7)	8595 (35.5)		
MD	(*n* = 1)	(*n* = 149)		
ISS			< 0.001	
Median (IQR)	9 (11)	13 (11)		
Categories			< 0.001	
1—9	162 (53.1)	10,611 (44.2)		
10—15	57 (18.7)	3584 (14.9)		
16—24	62 (20.3)	5485 (22.8)		
≥ 25	24 (7.9)	4355 (18.1)		
MD	(*n* = 2)	(*n* = 308)		
GCS			< 0.001	
15—13	269 (89.1)	19,233 (80.2)		
12—9	14 (4.6)	1407 (5.9)		
$\le$ 8	19 (6.3)	3339 (13.9)		
MD	(*n* = 5)	(*n* = 364)		
Hospital course and outcome data				
Admission to TICU			0.131	
Yes	38 (12.4)	3776 (15.5)		
TICU LOS, days			0.431	
Median (IQR)	12.5 (19)	14 (19)		
MV required			0.031	
Yes	46 (15.0)	4852 (19.9)		
MV, days			0.945	
Median (IQR)	11 (21)	10 (18)		
Hospital LOS, days			0.211	
Median (IQR)	8 (12)	9 (14)		
MD	(*n* = 5)	(*n* = 175)		
In-hospital mortality			0.045	
Alive	285 (92.8)	21,735 (89.3)		
Dead	22 (7.2)	2608 (10.7)		

*MD* missing data; *IQR* interquartile range; *PR* Puerto Rico; *MVA* motor vehicle accident; *GSW* gunshot wound; *SW* stab wound; *ABD* arterial base deficit; *SBP* systolic blood pressure; *ISS* Injury Severity Score; *GCS* Glasgow Coma Scale; *TICU* trauma intensive care unit; *LOS* length of stay; *MV* mechanical ventilation. Comparisons were assessed using data from the patients’ first admission, except for in-hospital mortality, where the last admission was used

Regarding the type of injury, penetrating trauma was more common among recidivists (39.5% vs. 26.9%; *p* < 0.001). The leading mechanism of injury overall was motor vehicle accident (MVA), occurring mostly in the non-recidivism group (36.8% vs. 35.2%). Nevertheless, there was a greater proportion of gunshot wounds (GSWs) (30.3% vs. 19.0%) and stab wounds (SWs) (9.5% vs. 7.7%) among recidivist trauma patients (*p* < 0.001). Violence-related injuries were the most prevalent mechanisms of trauma among recidivists (44.6% vs. 29.4%; *p* < 0.001).

As to vital signs (i.e., breathing, SBP, temperature, and heart rate) and ABD, there were no statistically significant differences (*p* > 0.05) between the study groups. Similarly, the proportion of patients admitted to TICU, TICU LOS, MV days, and hospital LOS were comparable between recidivist and non-recidivist patients (*p* > 0.05). However, the relative frequency of patients requiring MV was lower for those with more than one trauma-related admission than for their counterparts with only one trauma-related admission (15.0% vs. 19.9%; *p* = 0.031).

When evaluating injury severity markers, recidivist subjects were significantly less likely to have an ISS above or equal to 25 (7.9% vs. 18.1%; *p* < 0.001) and a GCS below or equal to 8 (6.3% vs. 13.9%; *p* < 0.001) relative to their non-recidivist counterparts. In-hospital mortality was also lower among patients with more than one trauma-related admission (7.2% vs. 10.7%; *p* = 0.045).

The results of the multivariate regression analysis revealed factors independently associated with trauma recidivism, as displayed in Table 2. However, none of the interaction terms tested in the model had a significant effect. Males were 3.88 (95% CI: 2.21–6.80) times more likely to suffer from a second injury episode; and individuals aged between 15 and 24 years old were 3.80 (95% CI: 2.24–6.46) times more likely. Moreover, patients with public health insurance had a 51% [adjusted odds ratio (AOR) = 1.51; 95% CI: 1.12–2.03] increased risk of experiencing another injury. An ISS above or equal to 25 (AOR = 0.44; 95% CI: 0.28–0.68) and a GCS below or equal to 8 (AOR = 0.56; 95% CI 0.34–0.92), conversely, were demonstrated to be independent protective factors from a second traumatic event.

**Table 2:** Univariate and multivariate regression analyses of sociodemographic characteristics, injury profile, and hospital course associated with trauma recidivism

Characteristic	UOR (95% CI)	AOR (95% CI)
Sex		
Male	4.42 (2.58–7.57)	3.88 (2.21–6.80)
Female	Ref	Ref
Age, years		
< 15	1.60 (0.72–3.54)	1.44 (0.62–3.35)
15—24	5.08 (3.13–8.24)	3.80 (2.24–6.46)
25–34	3.32 (2.00–5.52)	2.38 (1.37–4.14)
35–44	3.29 (1.93–5.63)	2.55 (1.44–4.49)
45–54	3.49 (2.02–6.05)	3.01 (1.70–5.31)
> 54	Ref	Ref
Insurance status		
Private insurance	Ref	Ref
Public insurance	1.94 (1.51–2.48)	1.51 (1.12–2.03)
Uninsured	1.52 (1.05–2.19)	1.03 (0.67–1.57)
Mechanism of injury		
MVA	2.42 (1.39–4.24)	1.57 (0.89–2.79)
GSW	4.04 (2.30–7.10)	1.72 (0.92–3.20)
SW	3.10 (1.63–5.87)	1.33 (0.66–2.66)
Falls	1.71 (0.92–3.21)	1.23 (0.64–2.38)
Pedestrians	Ref	Ref
Others	2.77 (1.46–5.23)	1.59 (0.81–3.10)
ISS		
≥ 25	0.39 (0.25–0.59)	0.44 (0.28–0.68)
< 25	Ref	Ref
GCS		
≤ 8	0.42 (0.26–0.66)	0.56 (0.34–0.92)
> 8	Ref	Ref
MV required		
Yes	0.71 (0.52–0.97)	0.95 (0.67–1.34)
No	Ref	Ref

*UOR* unadjusted odds ratio; *CI* Confidence Interval; *AOR* adjusted odds ratio; *Ref* reference category; *MVA* motor vehicle accident; *GSW* gunshot wound; *SW* stab wound; *ISS* Injury Severity Score; *GCS* Glasgow Coma Ccale; *MV* mechanical ventilation

### Sub-analysis: comparing first and second injury episodes among recidivists

**Fig. 1:**
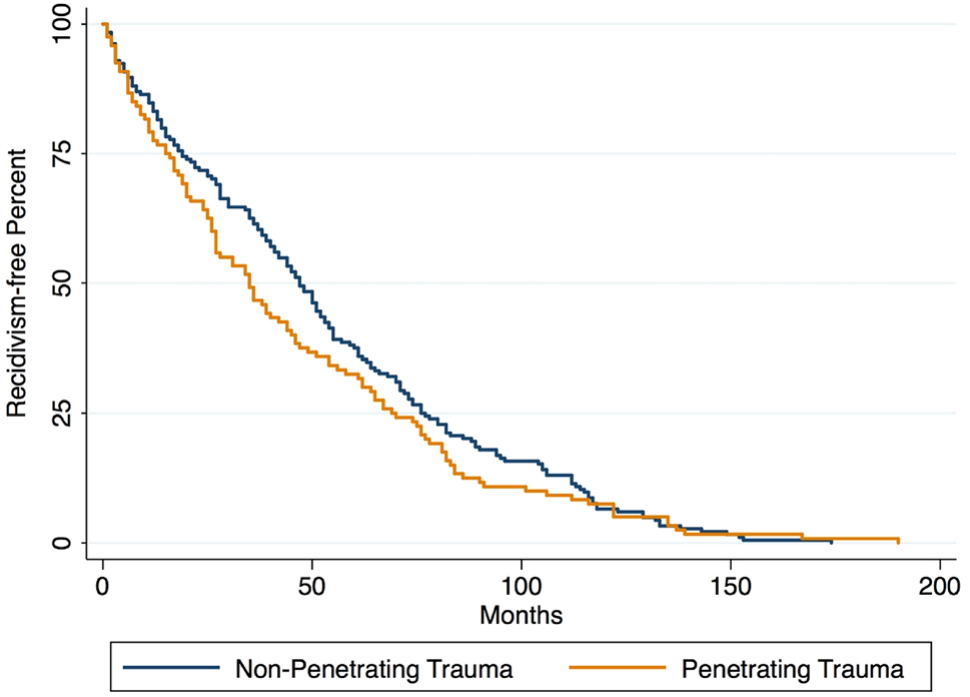
Comparison of Time to Reinjury between Recidivist Patients whose First Traumatic Event was Non-Penetrating and Recidivist Patients whose First Traumatic Event was Penetrating

The median [interquartile range (IQR)] time to reinjury for recidivists overall was 42 (59) months. In the mechanism-stratified analysis, the median (IQR) time to reinjury for patients presenting non-penetrating trauma during their first admission was 47 (57) months, whereas the median (IQR) time to reinjury for patients presenting penetrating trauma was 35 (54) months, as shown in Fig. 1. This difference was demonstrated to be marginally significant (*p* = 0.073).

**Fig. 2:**
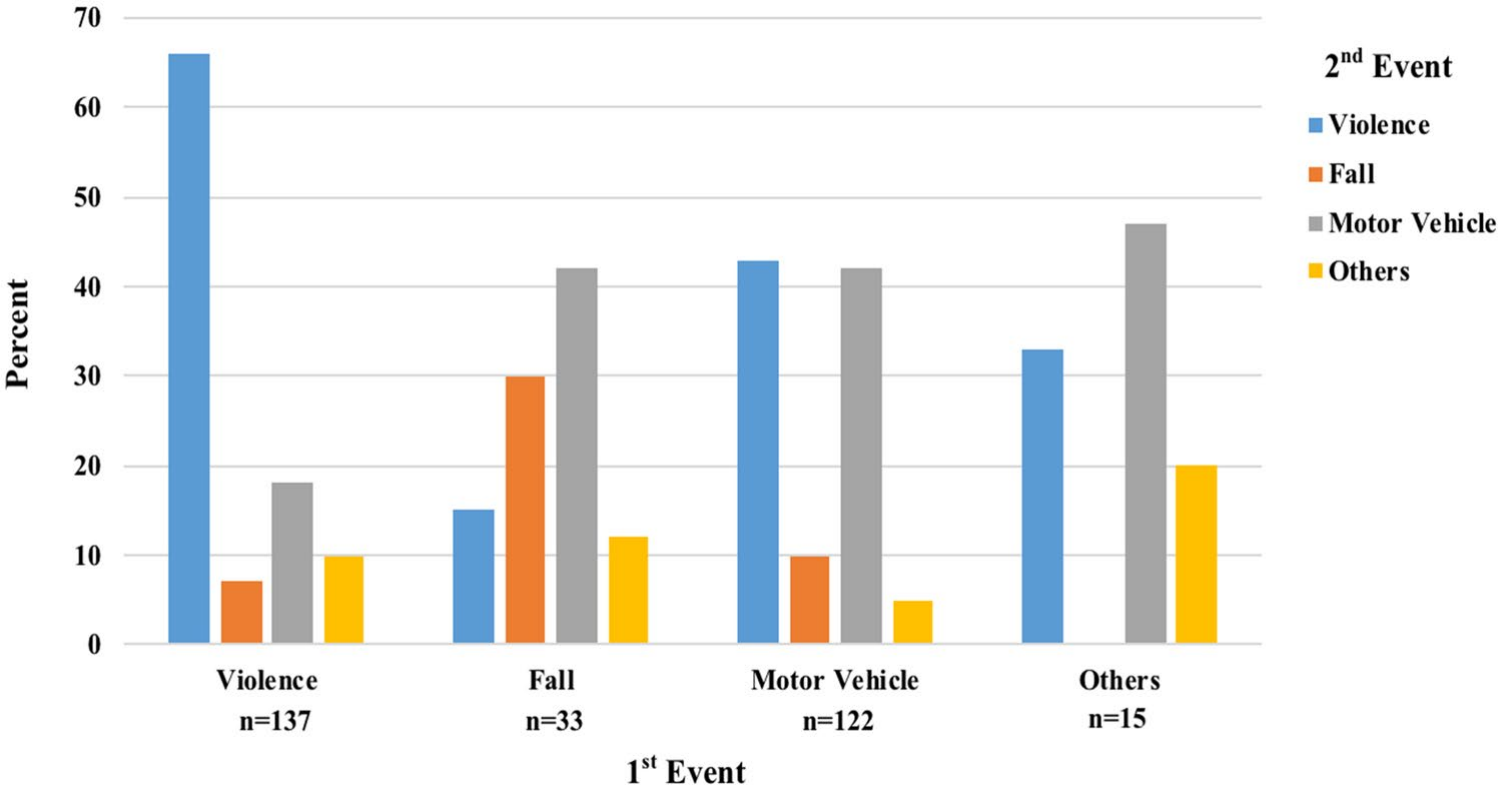
Distribution of Injury Mechanisms of the 2nd Event, Stratified by Injury Mechanisms of the 1st Event, among Recidivist Patients Admitted to the Puerto Rico Trauma Hospital

Recidivist subjects experienced more violence-related injuries (44.6% vs. 49.8%) during their second admission than during their first one. Motor vehicle-related injuries, including individuals run over, exhibited the opposite result (39.7% vs. 31.3%; *p* = 0.027). Of recidivist trauma patients with violence-related injuries during their first admission, 65.7% of the patients returned to the hospital for the same type of injury (see Fig. 2).

Additionally, the second injury episode was often more severe than the first, as the proportion of patients with an ISS above or equal to 25 increased (7.9% vs. 14.1%; *p* = 0.022). The number of subjects with GCSs below or equal to eight also showed a marginally significant increase (6.3% vs. 10.5%; *p* = 0.064). Furthermore, the need for MV was significantly greater (15.0% vs. 21.8%; *p* = 0.026) and the hospital LOS was marginally longer [median (IQR): 8 (12) days vs. 9 (16) days; *p* = 0.061] during the second trauma-related admission. Admissions to TICU were similar in both traumatic events (*p* > 0.05). Table 3 describes the comparison of injury profile and hospital course between the first and second injury episode in recidivist trauma patients.

**Table 3:** Comparison of injury profile and hospital course between the 1st and 2nd traumatic event in recidivist trauma patients admitted to the Puerto Rico trauma hospital

Characteristic	1st traumatic event *n* (%)	2nd traumatic event *n* (%)	*p* value
Injury-related data			
Type of injury A			0.127
Penetrating	121 (39.5)	137 (44.8)	
Type of Injury B			0.027
Violence related	137 (44.6)	153 (49.8)	
Fall	33 (10.8)	31 (10.1)	
Motor vehicle related	122 (39.7)	96 (31.3)	
Others	15 (4.9)	27 (8.8)	
ISS			0.082
Median (IQR)	9 (11)	10 (8)	
Categories			0.022
≥ 25	24 (7.9)	43 (14.1)	
GCS			0.064
$\le$ 8	19 (6.3)	32 (10.5)	
Hospital course			
Admission to TICU			0.104
Yes	38 (12.4)	52 (16.9)	
MV required			0.026
Yes	46 (15.0)	67 (21.8)	
Hospital LOS, days			0.061
Median (IQR)	8 (12)	9 (16)	

*1st* first; *2nd* second; *ISS* Injury Severity Score; *IQR* interquartile range; *GCS* Glasgow Coma Scale; *TICU* trauma intensive care unit; *MV* mechanical ventilation; *LOS* length of stay

## Discussion

The present study primarily sought to compare recidivist and non-recidivist trauma patients in terms of their sociodemographic characteristics, injury profile, and hospital outcomes. In the PRTH, 1.4% of the population experienced recidivism. Previously, rates of recidivism had been found to differ from 0.38% to 44% depending on location [5, 6]. This is a considerable variation that could be explained by several studies including less severe injuries; by including evaluations made in the periphery; and by excluding mechanisms of injury unrelated to violence. A male majority among these patients in our institution is also consistent with the existing literature [5, 12, 17, 18]. Interestingly, the descriptive analysis suggests that a larger proportion of recidivists present at an earlier age, with 40% of cases being reported among the 15–24 age group. This was comparable to findings published by Strong et al., in which 37.3% of recidivists were aged 18–29 years old [13], distinct from other pieces that identified recidivists to be over 30 years old [5, 10, 19]. Our population of recidivists frequently used public health insurance, contrast to other populations that are predominantly uninsured [9, 12, 18]. A possible explanation for this discrepancy is the accessibility to public health insurance among the population served by our institution. When admitted to our hospital, uninsured patients are often offered the opportunity to acquire public health insurance with assistance from social workers.

In PRTH, the leading mechanism of injury was MVA, notably among non-recidivists, likewise to other findings throughout the years [5, 8, 9, 17]. Violence-related injuries overall occurred largely among recidivists, as previously described [8, 19]. However, the percentage of violence-related injuries among recidivists in PRTH (44.6%) was considerably higher than the 25% seen by Dixon et al. and the 15% documented by Erdogan et al. [8, 19]. Furthermore, a previous study revealed recidivist patients were likely to present with the same mechanism of injury in the first and second traumatic events, with rates of up to 75% [5]. In our population, 65.7% of recidivists presenting with violence-related injuries during the first event returned for the same type of injury. Although McCoy et al. used different classifications for violent injuries, it was reported that 34% of GSW/SW victims and 37% of assault victims tend to recur with the same mechanism of injury [18]. Further analysis suggests PRTH recidivists suffer from more violence-related injuries on their second event when compared to their first event; whilst the contrary occurred with motor vehicle-related trauma.

The ISS, GCS, MV requirement, and mortality have been considered questionable due to limited evaluation. Consistent with our ISS results, Caufield et al. demonstrated recidivists suffered less severe injuries than non-recidivists, as did Dixon et al. and Erdogan et al. [5, 8, 19]. Regarding the GCS, our recidivists were less likely to have a severe state. In a 2007 study, Toschlog et al. documented a higher GCS in recidivist patients as compared to their non-recidivist counterparts, whereas Caufield et al. found no such difference [5, 20]. In addition to these severity markers, the PRTH patients with one trauma related admission had a greater need for MV than their recidivist counterparts. Previously, no differences in in-hospital mortality based on the recidivism status of patients were detected in many scientific works [8, 19, 20]. Yet, our analysis suggests PRTH recidivists were less likely to die during their hospital stay. Kwan et al. described a similar finding, with non-recidivists suffering from higher in-hospital mortality [12].

The median time to reinjury for recidivist trauma patients was 42 months. A study of a Canadian recidivist population exhibited an average 41 months to reinjury, which is closest to our marks [19]. Closer to one of the first reinjury analyses, with a median time of 7.9 months, Kaufmann et al. demonstrated a median time of approximately 10 months to reinjury [14, 21]. There is a wide range of times to reinjury reported in the scientific literature, possibly due to either limitations or wide access to healthcare center locations of various levels. Moreover, the time to reinjury could vary according to the type of trauma, as alluded in our institution. The time to reinjury for non-penetrating trauma and penetrating trauma differed by about a year, with penetrating injuries occurring earlier. A possible explanation is that non-penetrating trauma, although commonly involves a third party, has more elements under the control of the possible recidivist. These factors may include seatbelt usage, traffic vigilance, prevention of falls through avoidance of clutter at ground level, stairs, and protected ladder usage, among others. Penetrating trauma, however, involves third party behavior commonly beyond the victim’s control.

As for predictors of reinjury, male sex was a significant risk factor for all forms of recidivism, consistent with literature on violent recidivism [11, 21, 22]. Furthermore, the 15–24-year-old patients in PRTH were found to be almost four times as likely to be recidivists. This was a stronger association than previously reported by Algham et al., in which 18–25-year-old patients were almost twice as likely to be recidivists [23]. Public health insurance was also independently associated with an increase in all forms of reinjury, as depicted in other literature [23]. Interestingly, however, a severe GCS or ISS could be a protective factor for our population. Patients with a GCS less than or equal to eight or an ISS greater than or equal to 25 were half as likely to become recidivists, thereby serving as potential protective factors. In other general trauma recidivism studies, no predictive effect has been found for the GCS or ISS [10]. A violent recidivism study by Kaufmann et al. also conveyed this lack of value [21]. Yet, Nygaard et al. stated a lower ISS was a predictor of violent recidivism [22]. The protective effect found in the severity markers could be due to consequential lifestyle or functional impairments which led to avoidance of the situation or environment, precautionary measures instilled, possible death due to trauma before a second admission, or loss of follow-up due to emigration. Nevertheless, further investigation of long-term mortality and functional impairments of these patients, including all mechanisms of trauma, would be of benefit to clarify these risk or protective factors.

The early identification of potential recidivist trauma patients by hospital personnel, and an in-depth history assessment, including substance abuse and psychiatric disorders, could expand our knowledge base and promote investigation. This could in turn be used to tailor prevention platforms targeting road safety, violence, and mental health, among others. Previously, health care-based violence interventions directed towards recidivism prevention have shown some improvements [13]. Moreover, the establishment of community based, mental health, and substance abuse treatment programs could be useful before the recurrence. Finally, time to reinjury supplies valuable information on the window of prevention to establish these methods efficiently and effectively.

There were limitations to rendering a definitive picture of recidivism. Several records were lacking information, whereas others could have had variables erroneously evaluated. Most files were missing variables related to substance use, including alcohol use, which led these to be excluded from further study. Self-inflicted injuries and repeated attempted suicides were also not explicitly included in the study. Although the aforementioned categories represent a very small fraction of the population, exclusion of them could contribute to bias in our findings. Recurring patients may have also had their information incorrectly entered under a new record number and, consequently, been identified mistakenly as a non-recidivist. Additionally, the island has had a high emigration rate throughout the years, suggesting patients leave before a possible recurrence. Recidivists could have also arrived to institutions in the periphery and have been effectively managed. However, this is less likely due to high referral rate to our trauma center, the only tertiary level II center on the island. Death of patients on scene either early after the initial injury, or due to comorbidity, could have affected the results and information available. Moreover, some recurring patients might have not been identified because the follow-up period was not drawn out enough for such an event to occur. Because of this, there could have been a misrepresentation of information and, subsequently, in the classification of recidivists and non-recidivists.

## Conclusion

Recidivists are more likely to be male, present at an earlier age (15–24-year age group) and use public health insurance. Additionally, recidivists were more likely to suffer violence-related injuries, suffer less severe injuries, be evaluated with a higher GCS, and have less need for MV. This PRTH subgroup also exhibited less in-hospital mortality. Comparing the first and second traumatic incidents of PRTH recidivists, findings disclosed more violence-related injuries on their second occurrence; whilst the contrary occurred with motor vehicle-related trauma. Second traumatic injuries were inclined to be graver than the first episodes, with a decline in neurological status, increasing severity scores, a marginally longer hospital stay, and greater requirement for MV. Risk factors of reinjury include the male sex, being 15–24 years old, and possessing public health insurance. However, a severe GCS or ISS could be protective factors. The median time to reinjury was 42 months, with penetrating trauma reoccurring about a year earlier than non-penetrating trauma, giving a window of opportunity for intervention. Future studies could be directed toward investigating long-term mortality, the evaluation of deaths on scenes, and verifying outcomes or presence of possible recidivists in surrounding hospitals. The usage of this information could promote effective interventions and awareness around the existing problem.
